# Exercise Preconditioning Promotes Autophagy to Cooperate for Cardioprotection by Increasing LC3 Lipidation-Associated Proteins

**DOI:** 10.3389/fphys.2021.599892

**Published:** 2021-05-05

**Authors:** Dong-Feng Wan, Shan-Shan Pan, Yi-Shan Tong, Yue Huang

**Affiliations:** School of Kinesiology, Shanghai University of Sport, Shanghai, China

**Keywords:** LC3, autophagy-related gene 4B, autophagy-related gene 7, autophagy-related gene 3, exercise preconditioning, cardioprotection

## Abstract

The cardioprotection of exercise preconditioning (EP) has been well documented. EP can be divided into two phases that are the induction of exercise preconditioning (IEP) and the protection of exercise preconditioning (PEP). PEP is characterized by biphasic protection, including early exercise preconditioning (EEP) and late exercise preconditioning (LEP). LC3 lipidation-mediated autophagy plays a pivotal role in cardioprotection. This study aimed to investigate the alterations of LC3 lipidation-associated proteins during EP-induced cardioprotection against myocardial injury induced by exhaustive exercise (EE) was used in a rat model of EP. These rats were subjected to an intermittent exercise consisting of four periods, with each period including 10 min of running at 30 m/min and 0% grade (approximately 75% VO_2max_) followed by 10 min of intermittent rest. A model of EE-induced myocardial injury was developed by subjecting rats to a consecutive running (30 m/min, 0% grade) till exhaustion. Following EEP, the colocalization of LC3 with Atg7 was significantly increased, and LC3-I, LC3-II, LC3-II/LC3-I, Atg7, Atg4B, and Atg3 levels were significantly increased. Atg7, Atg4B, and Atg3 mRNAs were all significantly upregulated, and LC3 mRNAs tended to be higher. Following LEP, Atg4B, and Atg3 levels were significantly increased. Atg7, Atg4B, and Atg3 mRNAs were all significantly upregulated, and LC3 mRNAs tended to be higher. A group of rats were subjected to EEP followed by EE, and the co-localization of LC3 with Atg7 was significantly increased, while LC3-I, LC3-II, LC3-II/LC3-I, Atg7, Atg4B, and Atg3 levels were also significantly increased. Moreover, there was a significant increase in the co-localization of LC3 with Atg7, LC3-I, LC3-II, Atg7, and Atg4B levels during LEP followed by EE. The formation of autophagosome during LEP followed by EE may have been weaker than that during EEP followed by EE due to the lower lipidation of LC3. EP may promote autophagy to maintain cell homeostasis and survival, which cooperates for cardioprotection of alleviating exhaustive exercise-induced myocardial injury by increasing LC3 lipidation-associated proteins. There is a difference between EEP and LEP in terms of the mechanisms of cardioprotection afforded by these respective conditions. The positive regulation of transcription and translation level of LC3 lipidation-associated proteins may all be involved in the mechanism of EEP and LEP, while compared with LEP, the regulation of translation level of EEP is more positively to promote autophagy.

## Introduction

The belief that physical exercise promotes optimal health and is integral in the prevention and treatment of many medical conditions is burgeoning, but this does not mean that all exercise is good for health. Regular exercise can reduce the risk of cardiovascular events and is clinically useful for cardioprotection against myocardial ischemia/reperfusion (I/R) injury (Borges and Lessa, 2015; Penna et al., 2020). However, excessive exercise can also cause myocardial injury. Evidence have demonstrated that even a single bout of high-intensity intermittent exercise is capable of forming a “preconditioned” cardioprotective phenotype against myocardial ischemic injury (Domenech et al., 2002; Parra et al., 2015). Exercise preconditioning (EP) refers to repeated high-intensity, intermittent aerobic exercise that can lead to myocardial relative or absolute ischemia-hypoxia, inducing endogenous cardioprotection to protect myocardial tissues against subsequent sustained ischemic-hypoxic injury (Domenech et al., 2002; Kavazis, 2009; Yuan et al., 2018). EP is similar to ischemic preconditioning (IP) to provide cardioprotection against myocardial I/R injury. EP can be divided into two phases that are the induction of exercise preconditioning (IEP) and the protection of exercise preconditioning (PEP). IEP refers to the process of inducing endogenous cardioprotection through repeated high-intensity, intermittent aerobic exercise, and PEP refers to the process of promoting endogenous cardioprotection after IEP (Li et al., 2019; Liu and Pan, 2019). PEP is characterized by biphasic protection, including early exercise preconditioning (EEP) and late exercise preconditioning (LEP). Specifically, EEP occurs immediately after the end of IEP and lasts for 1–3 h (Domenech et al., 2002; Kavazis, 2009), while LEP appears 24 h after the end of IEP and lasts for 24–72 h (Domenech et al., 2002; Thijssen et al., 2018).

The mechanism responsible for EP-induced cardioprotection is a multifactorial process, and the cardioprotective phenotype may be associated with multiple factors (Powers, 2017). Autophagy is a biological process in which cells capture aged and damaged proteins or organelles to be degraded or recycled within lysosomes (Gottlieb and Mentzer, 2013; Halling and Pilegaard, 2017). Autophagy can be activated or elevated to maintain cell homeostasis and survival under exercise, hypoxia, and other stress conditions, which is an endogenous protective mechanism against myocardial injury (Gottlieb and Mentzer, 2013). Studies have shown that ischemia-hypoxia can induce autophagy under different stress conditions (Gottlieb et al., 2009). Beclin1 is a marker protein for inducing autophagy. He et al. (2012) reported that myocardial autophagy induced by treadmill exercise was related to the induction of Beclin1, and the conversion of the non-lipidated form of microtubule-associated protein 1 light chain 3 (LC3/MAP1LC3), LC3-I, to the autophagosome-membrane-associated lipidated form, LC3-II, as well as the degradation of autophagy substrate protein p62, demonstrating that autophagy plays a pivotal role in exercise-induced cardioprotection.

Autophagy can be divided into four processes, and its core process is the formation of autophagosome (Noda and Inagaki, 2015). The formation of autophagosome is inseparable from the regulation of LC3 lipidation-associated proteins. LC3 is a marker protein for the formation of autophagosome, which involves the conversion of LC3-I to LC3-II. LC3-II levels and the LC3-II/LC3-I ratio can be considered indicators to reflect the level of autophagy (Lee et al., 2017). The precursor form of LC3 is truncated by autophagy-related gene 4B (Atg4B) to form LC3-I, and is formed into LC3-II by the action of E1 and E2-like enzymes autophagy-related gene 7 (Atg7) and autophagy-related gene 3 (Atg3; Gottlieb et al., 2015). Atg7 bound to LC3-I is important for autophagosomal biogenesis (Pattison et al., 2011). Increased autophagic synthesis and decreased lysosomal function are responsible for upregulation of LC3 levels (Pattison et al., 2011; Klionsky et al., 2016). p62/SQSTM1 is likely to play a role for lysosomal function as an autophagy receptor protein. In this study, we hypothesize that EP may provide a favorable cell survival environment to protect the heart from exhaustive exercise (EE)-induced injury *via* autophagy. In order to verify the above hypothesis, we focused on LC3 lipidation-mediated autophagy for cardioprotection by studying the changes of LC3, Atg4B, Atg7, Atg3, and p62 during EP. To decipher the role of LC3 lipidation-associated proteins during EP will provide an in-depth reference for the research on cardioprotective mechanisms of EP, and lead a potential prospect in the prevention and treatment of many medical and physical conditions.

## Materials and Methods

### Experimental Animals

Healthy male Sprague-Dawley rats (*n* = 120), age 8 weeks (Jie Si Jie Laboratory Animal Co. Ltd., Shanghai, China), were fed at constant temperature (22–24°C), humidity (40–70%), and with a 12-h light/dark period. The rats weighed, on average, 203 ± 7 g. All of the animal experiments were performed in accordance with Laboratory animal-Guideline for ethical review of animal welfare (GB/T 35892-2018, China) and approved by Ethics Committee of Science Research in Shanghai University of Sport, China.

### Experimental Protocol

**Figure 1:**
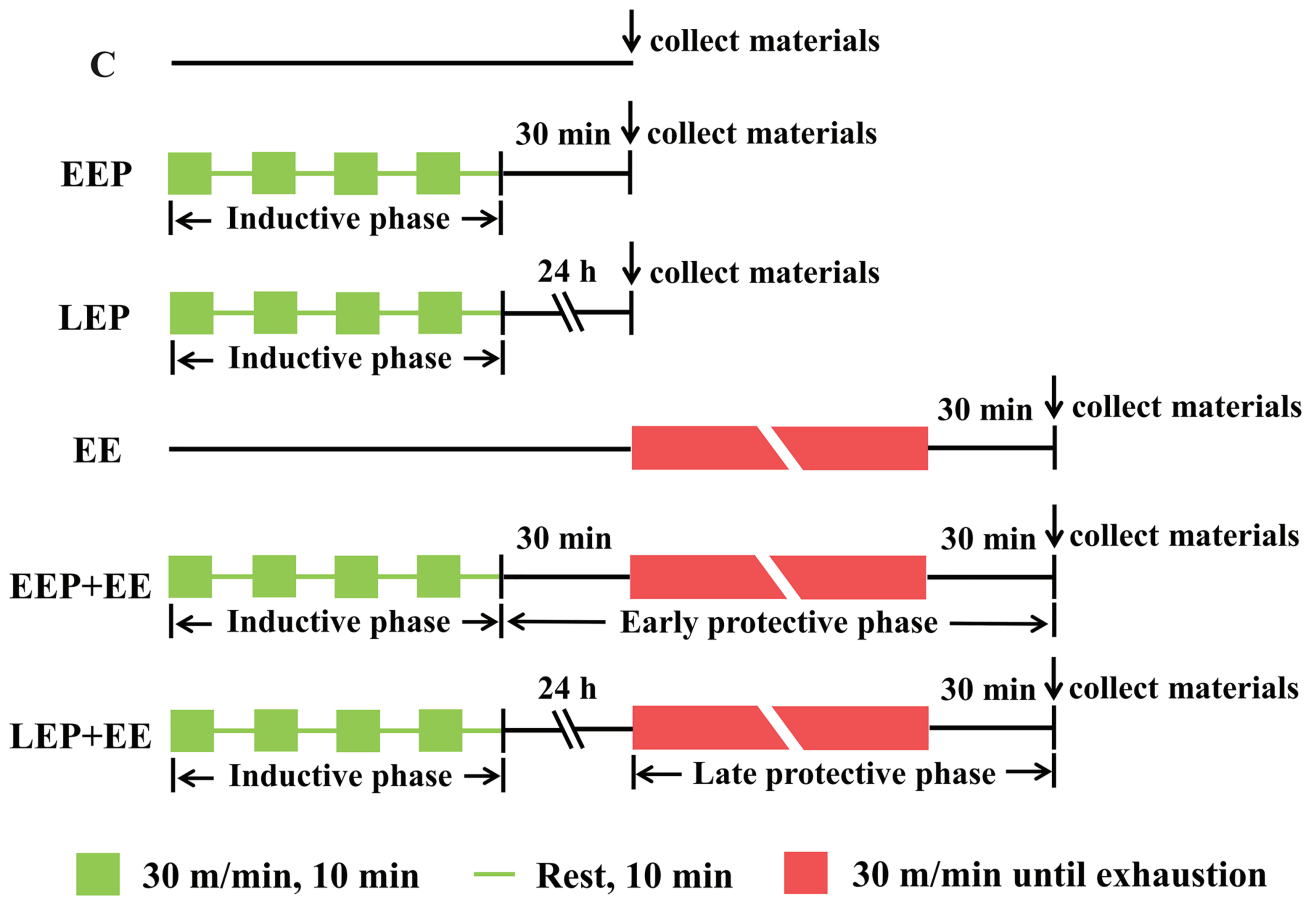
Exercise protocol. C, group control; EEP, group early exercise preconditioning; LEP, group late exercise preconditioning; EE, group exhaustive exercise; EEP + EE, group EEP plus exhaustive exercise; and LEP + EE, group late exercise preconditioning plus exhaustive exercise.

All of the rats were acclimatized to treadmill training, which included 5 consecutive days of treadmill exercise (15 m/min, 10 min/day, and 0% grade). After the treadmill exercise, the rats were randomly assigned to six experimental groups (*n* = 20 per group). Group C was control rats placed on the treadmill without any exercise intervention. Group EEP was challenged with intermittent exercise of four repeated cycle periods, each consisting of a 10-min run at 30 m/min and 0% grade (approximately 75% VO_2max_; Shepherd and Gollnick, 1976), with intermittent rest for 10 min. This group was used to investigate the changes of LC3 lipidation-associated proteins during IEP and EEP. Group LEP was treated similarly to group EEP, except for the sampling time. This group was used to investigate the changes of LC3 lipidation-associated proteins during IEP and LEP. Group EE was designed as an exhaustive exercise-induced myocardial injury model, and these rats were subjected to consecutive running (30 m/min, 0% grade) till exhaustion. The standard for rats run to exhaustion was the loss of righting reflex. The rats were unable to right themselves when placed on their backs. Group EEP + EE (EEP plus exhaustive exercise) performed the same running exercise as group EEP followed by exhaustive exercise after 30 min, and this group was used to explore the early cardioprotective effects of EP against myocardial injury induced by exhaustive exercise. Group LEP + EE (LEP plus exhaustive exercise) performed the same running exercise as group LEP followed by exhaustive exercise after 24 h, and this group was used to explore the late cardioprotective effects of EP against myocardial injury induced by exhaustive exercise. The EP experimental protocol designed was based on previous studies (Parra et al., 2015; Yuan and Pan, 2018; Yuan et al., 2018). All of the rats in exercise groups were sacrificed 30 min following exercise, except for the group LEP rats, which were sacrificed 24 h after IEP (Figure 1).

### Chromotrope-2R Brilliant Green Staining

Normal myocardium was stained green by chromotrope-2R brilliant green (C-2R BG) staining. Chromotrope 2R has a strong affinity for ischemic-hypoxic myocardium and stained these cells red. Slides were dewaxed to water and stained with a chromotropic acid 2R solution for 10 min. Then, they were differentiated in a 0.2% acetic acid solution for 2–3 min, and added to 5% bright green glacial acetic acid dye for 5–10 min. Eventually, the slides were cleared by xylene and observed with a microscope (BX53, Olympus, Japan; Lu et al., 2018). A total of 25 images were collected using a microscopic digital camera (DP80, Olympus, Japan) for statistical analysis from each group. Each visual field was analyzed using Image-Pro Plus 6.0 analytic software (Media Cybernetics, Silver Spring, United States). The measurement parameters were selected as integral optical density (IOD) and positive area of ischemic-hypoxic myocardium, and the mean optical density (MOD) was used to show the damage degree of single positive areas of ischemic-hypoxic myocardium (MOD = IOD/positive area).

### Chemiluminescence

Chemiluminescence, a double site immunoassay, was used to quantify plasma cardiac troponin I (cTnI) levels using the Access AccuTnI + 3 kit (Beckman Coulter, United States, A98264) based on a multi-point calibration curve. The monoclonal anti-cTnI antibody was combined with alkaline phosphatase and added to the reaction tube, along with buffer and samples. The cTnI anti-cTnI antibody was combined in the solid state and allowed to interact in the reaction tube. The chemical luminescent substrate, Lumi-Phos*530, was added to the reaction tube, and the light produced by the reaction was detected with a photometer. The amount of light detected was indicative of the concentration of cTnI in each sample.

### Electrocardiogram Acquisition and Analysis

The ECG signals of rats were collected and recorded using an ECG acquisition and analysis system (SP2006, Softron Beijing Biotechnology Co. Ltd., China). After intraperitoneal anesthesia with 0.4% pentobarbital sodium (4 mg/100 g body weight), the abdomens of rats were turned upward. The limb leads of animal ECG were connected to the limbs of rats fixed on an anatomical table, and then the ECG acquisition system was switched on to collect and record the ECG signals of each rat for 3 min. Myocardial ischemic-hypoxic injury was evaluated using the ST-segment and T-wave of an ECG (Tung, 2019).

### Immunofluorescence Staining

For immunofluorescence, we observed the extent of colocalization of LC3 and Atg7 and the localization of Atg4B and Atg3, respectively, in myocardium tissue. Paraffin sections were rehydrated in different gradients of ethanol, and we then added complex digestive juices for 5 min, and incubated the slides with 5% goat serum for 1 h at room temperature to block nonspecific binding of antibodies. For the colocalization of LC3 and Atg7, slices were incubated with hybrid primary antibodies overnight at 4°C in wet boxes. The primary antibodies details are given as follows: LC3 (1:500, Sigma, L7543), Atg7 (1:500, Santa Cruz, sc-376212). This was followed by a 1 h incubation with the hybrid secondary antibody, Alexa Fluor 488-AffiniPure Donkey Anti-Rabbit IgG (H + L; 1:400, Jackson, 711-545-152) and Alexa Fluor 594-AffiniPure Donkey Anti-Mouse IgG (H + L) (1:400, Jackson, 715-585-151), and counterstaining with DAPI (1:1,500; BYT, C1002). LC3 was stained green, Atg7 was stained red, and the colocalization of LC3 and Atg7 was thus yellow. For the localization of Atg4B and Atg3, slices were incubated with primary antibodies against Atg4B (1:50, Novus, NBP2-41307) or Atg3 (1:500, Santa Cruz, sc-393660) overnight at 4°C in wet boxes. This was followed by incubation with Alexa Fluor 488-AffiniPure Donkey Anti-Rabbit IgG (H + L) secondary antibody (Jackson) or Alexa Fluor 594-AffiniPure Donkey Anti-Mouse IgG (H + L) secondary antibody (Jackson), respectively, for 1 h and counterstaining with DAPI (BYT). The experimental results were observed using a laser scanning confocal microscope (LSM700, Carl Zeiss, Germany).

### Western Blotting

The left ventricular myocardium of each rat was cut into many small tissues and added to RIPA buffer with PMSF and protease inhibitors to prevent protein degradation. The protein concentration of the lysate was then assessed by a bicinchoninic acid assay. Lysates were then separated by SDS-PAGE, followed by transfer to polyvinylidene fluoride membranes. Different voltages were selected based on the molecular weight of the protein of interest for transferring to membranes. Membranes were then blocked for 2 h at room temperature in 7% nonfat dried milk, and incubated overnight at 4°C with primary antibodies against Beclin1 (1:500, Santa Cruz, sc-48381), LC3 (1:500, Sigma, L7543), Atg7 (1:1,000, Cell Signaling, #8558), Atg4B (1:50, Novus, NBP2-41307), Atg3 (1:500, Santa Cruz, sc-393660), p62 (1:1,000, Cell Signaling, #23214), and GAPDH (1:5,000, Zen BioScience, 200306-7E4). Following incubation with primary antibodies, the membranes were probed with horseradish peroxidase (HRP) conjugated goat anti-rabbit IgG (H + L) secondary antibody (1:5,000; BYT, A0208) or goat anti-mouse IgG (H + L) secondary antibody (1:5,000; BYT, A0216), respectively, for 1 h at room temperature. The proteins were visualized using chemiluminescence reagent (Tanon™ High-sig ECL Western Blotting Substrate, Tanon, China) and measured by Image J analytic software (NIH, Bethesda, United States). The relative gray value of the above-mentioned protein is expressed as the ratio of their gray value relative to the gray value of GAPDH, indicating the level of these proteins.

### Quantitative Real-Time PCR

The left ventricular myocardium tissue from each rat was homogenized in Trizol reagent. RNA was isolated by chloroform phase separation and alcohol precipitation, and its concentration and purity was determined by UV absorption. cDNA synthesis was performed by the Thermo Scientific RevertAid First Strand cDNA Synthesis Kit (Thermo Fisher, United States, #K1622). Primers for LC3, Atg7, Atg4B, Atg3, and GAPDH were designed with Primer-BLAST (National Library of Medicine, Bethesda, United States). The primers are shown in Table 1. Real-time PCR (RT-PCR) was performed using FastStart Universal SYBR Green Master (Roche, Switzerland, 04913914001).

**Table 1:** Sequences of the primers.

Gene	Forward primer	Reverse primer
Atg3	5'-TGGAAGTGGCCGAGTACCTGAC-3'	5'-GCCATGTTGGACAGTGGTGGAC-3'
Atg7	5'-GTGTACGATCCCTGTAACCTAACCC-3'	5'‐ CGAAAGCAGAGAACTTCAACAGACT-3'
Atg4B	5'-CGAGAGCTTCCACTGCCAACAC-3'	5'-GAAGGCTGCTGTTCCACCAACTC-3''
LC3	5'-CGTCCTGGACAAGACCAAGTTCC-3'	5'-CAGGAGGAAGAAGGCTTGGTTAGC-3'
GAPDH	5'-GGAAAGCTGTGGCGTGAT-3'	5'-AAGGTGGAAGAATGGGAGTT-3'

### Statistical Analysis

Data were analyzed using the SPSS statistical software package (SPSS 17.0, IBM, United States), and were presented as mean ± SD. The values in each group were compared by one-way ANOVA, and a least significant difference (LSD) test was conducted on the basis of significant differences between the groups. Correlation analysis was based on the Bivariate of Correlate. Statistically significant difference was set at a value of *p* < 0.05.

## Results

### EP Induced Myocardial Autophagy in Response to Intermittent Ischemia-Hypoxia

**Figure 2:**
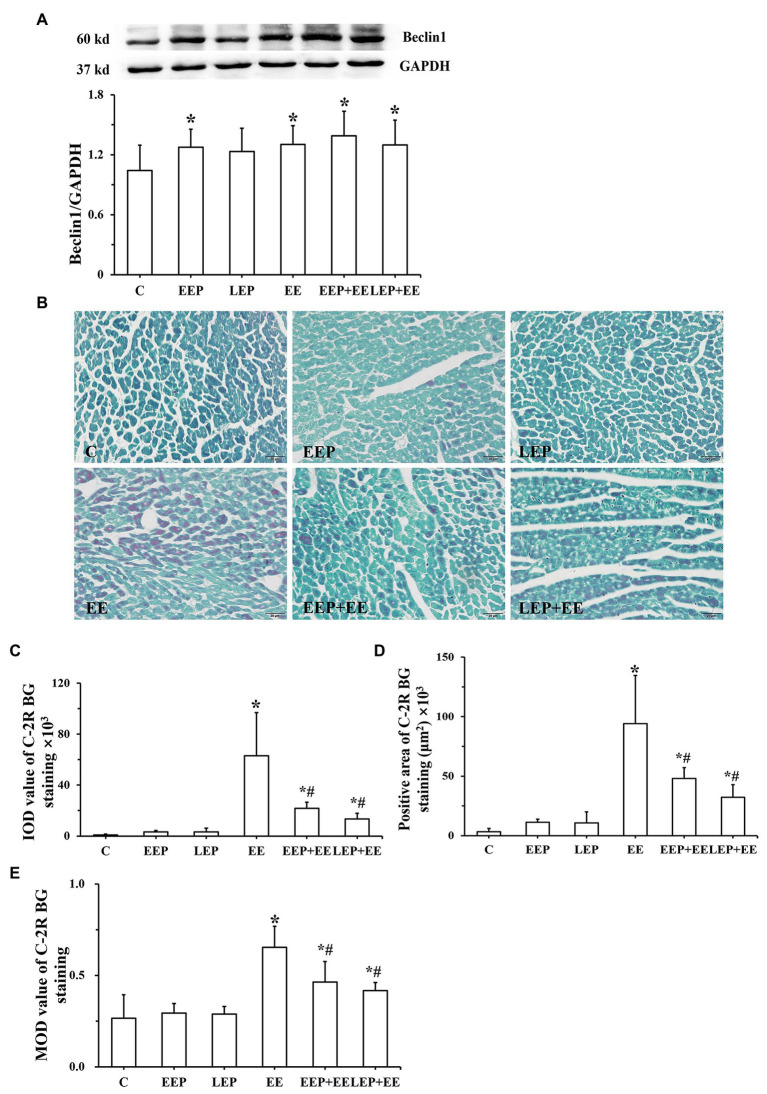
Expression of Beclin1 and degree of ischemia-hypoxia in different groups. **(A)** Western blot analysis of Beclin1 in different groups. **(B)** Chromotrope-2R brilliant green (C-2R BG) ischemia-hypoxia staining (bar = 100 μm). The C-2R BG-stained images showed that normal myocardium was stained green in contrast to the bright red of ischemic-hypoxic myocardium. **(C)** Integral optical density (IOD) of C-2R BG ischemia-hypoxia staining. **(D)** Positive area of C-2R BG ischemia-hypoxia staining. **(E)** Mean optical density (MOD) of C-2R BG ischemia-hypoxia staining. ^*^p < 0.05 vs. group C and ^#^*p* < 0.05 vs. group EE.

Western blot analysis of Beclin1 is shown in Figure 2A. These results demonstrated that compared with group C, Beclin1 levels were significantly higher in groups EEP, EE, EEP + EE, and LEP + EE (*p* < 0.05), and Beclin1 levels in group LEP tended to be higher (*p* > 0.05).

Chromotrope-2R brilliant green-staining images show that normal myocardium was stained green in contrast to the bright red of ischemic-hypoxic myocardium (Figure 2B). The myocardium from group C had green staining. Large areas of ischemic-hypoxic regions in group EE stained bright red, while there were few bright red regions in groups EEP + EE or LEP + EE. Image analysis (Figures 2C–E) further demonstrate that in contrast to group C, groups EE, EEP + EE, and LEP + EE had significantly higher IOD, positive area and MOD (*p* < 0.05), and IOD, positive area and MOD in groups EEP and LEP tended to be higher (*p* > 0.05). The IOD, positive area, and MOD in groups EEP + EE and LEP + EE were significantly decreased compared with those from group EE (*p* < 0.05).

**Figure 3:**
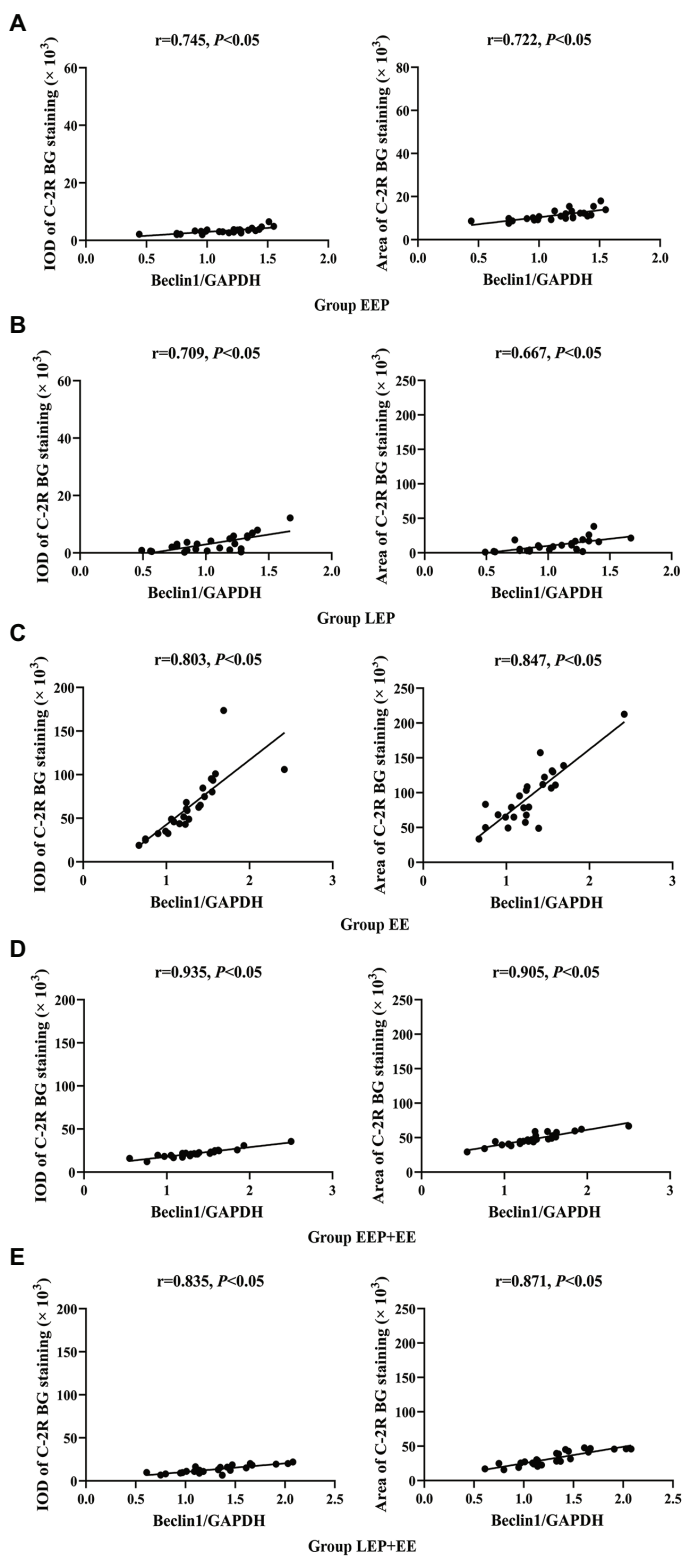
Correlation between ischemia-hypoxia and Beclin1 levels between groups. **(A)** Group EEP. **(B)** Group LEP. **(C)** Group EE. **(D)** Group EEP + EE. **(E)** Group LEP + EE.

Correlation analysis (Figures 3A–E) between the IOD or positive area of C-2R BG and Beclin1 show that ischemia-hypoxia can induce myocardial autophagy. In group EE, both IOD (*R* = 0.803) and positive area (*R* = 0.847) were positively correlated with Beclin1. In group EEP + EE, IOD (*R* = 0.935) and positive area (*R* = 0.905) were also positively correlated with Beclin1. In group LEP + EE, IOD (*R* = 0.835) and positive area (*R* = 0.871) were positively correlated with Beclin1. In group EEP, both the IOD (*R* = 0.745) and positive area (*R* = 0.722) were positively correlated with Beclin1. In group LEP, IOD (*R* = 0.709) and positive area (*R* = 0.667) were also positively correlated with Beclin1.

### Expression of LC3 Lipidation-Associated Proteins During IEP

**Figure 4:**
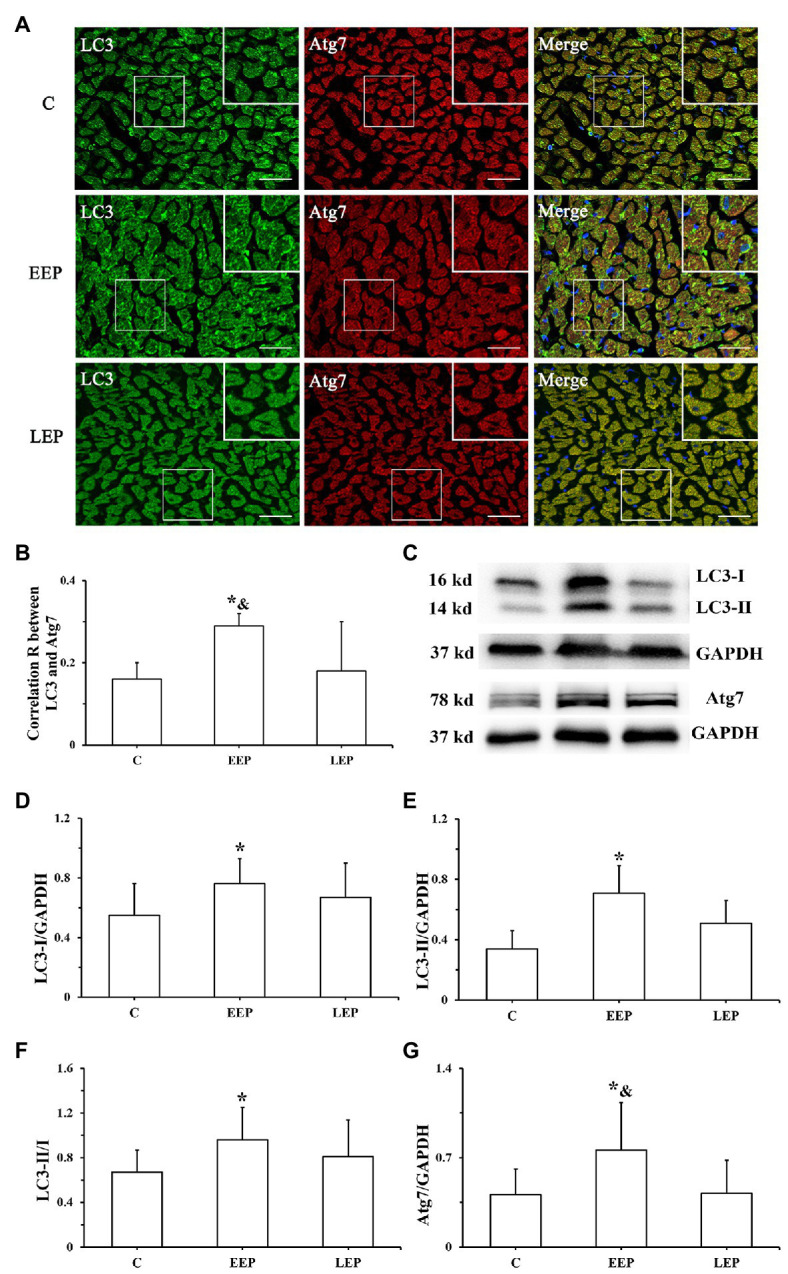
Expression of LC3 and Atg7 levels during EP. **(A)** Immunofluorescent staining of LC3 and Atg7 in cardiomyocytes (bar = 100 μm). LC3 immunofluorescence showed green puncta distributed in the cytoplasm. The immunofluorescence images of Atg7 had red puncta distributed in the cytoplasm, and the merged images yielded yellow puncta. **(B)** Colocalization of LC3 with Atg7 shown as a correlation R. **(C)** Western blot of LC3 and Atg7. **(D)** Western blot analysis of LC3-I in different groups. **(E)** Western blot analysis of LC3-II in different groups. **(F)** Western blot analysis of LC3-II/LC3-I in different groups. **(G)** Western blot analysis of Atg7 in different groups. ^*^p < 0.05 vs. group C; ^&^*p* < 0.05 vs. group LEP.

Immunofluorescence results (Figure 4A) show the colocalization of LC3 with Atg7 in the myocardium. LC3 immunofluorescence images displayed green puncta, including pro-LC3, LC3-I, and LC3-II, distributed in the cytoplasm. The immunofluorescence images of Atg7 had red puncta distributed in the cytoplasm, and the merged images displayed yellow puncta. Statistics of the colocalization of LC3 with Atg7 (Figure 4B) demonstrate that, compared with group C, the correlation R was significantly higher in group EEP (*p* < 0.05) in contrast to group LEP, which tended to be higher (*p* > 0.05). Compared with group LEP, the correlation R of group EEP was significantly higher (*p* < 0.05). Western blot analysis of LC3 and Atg7 is shown in Figures 4C–G. These results demonstrated that, compared with group C, LC3-I, LC3-II, LC3-II/LC3-I, and Atg7 levels were significantly higher in group EEP (*p* < 0.05), in contrast to the levels of group LEP, which tended to be only marginally higher (*p* > 0.05). The Atg7 levels of group EEP were significantly increased compared with group LEP (*p* < 0.05). The LC3-I, LC3-II, and LC3-II/LC3-I levels of group EEP tended to be higher compared with group LEP (*p* > 0.05).

**Figure 5:**
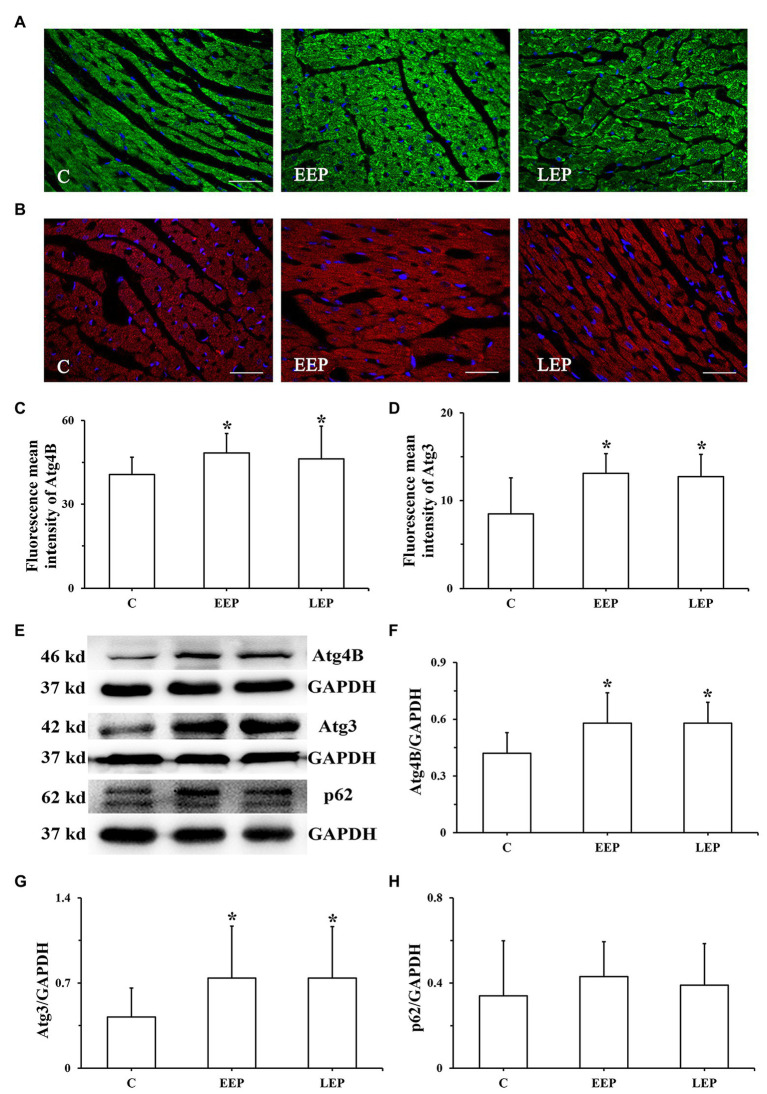
Expression of Atg4B, Atg3, and p62 levels during EP. **(A)** Immunofluorescence staining of Atg4B in cardiomyocytes (bar = 100 μm). Atg4B was stained green and had a scattered distribution in group C. **(B)** Immunofluorescence staining of Atg3 in cardiomyocytes (bar = 100 μm). Atg3 was stained red and had a scattered distribution in group C. **(C)** Fluorescence mean intensity of Atg4B immunofluorescent staining. **(D)** Fluorescence mean intensity of Atg3 immunofluorescent staining. **(E)**: Western blot of Atg4B, Atg3, and p62. **(F)** Western blot analysis of Atg4B in different groups. **(G)** Western blot analysis of Atg3 in different groups. **(H)** Western blot analysis of p62 in different groups. ^*^p < 0.05 vs. group C.

Immunofluorescence results (Figures 5A,B) show the expression patterns of Atg4B and Atg3 in myocardium. Atg4B immunoreactive products were visible as green spots, and Atg3 immunoreactive products were red spots, all of which were distributed in the cytoplasm. Analysis of Atg4B and Atg3 immunofluorescence results (Figures 5C,D) reveal that both Atg4B and Atg3 fluorescence mean intensity were significantly higher in groups EEP and LEP compared with those from group C (*p* < 0.05). Compared with group LEP, both Atg4B and Atg3 fluorescence mean intensity tended to be higher in group EEP (*p* > 0.05). Western blot analysis of Atg4B, Atg3, and p62 is shown in Figures 5E–H. In groups EEP and LEP, both Atg4B and Atg3 levels were significantly increased compared with those in group C (*p* < 0.05). The p62 levels in groups EEP and LEP tended to be higher compared with group C (*p* > 0.05).

**Figure 6:**
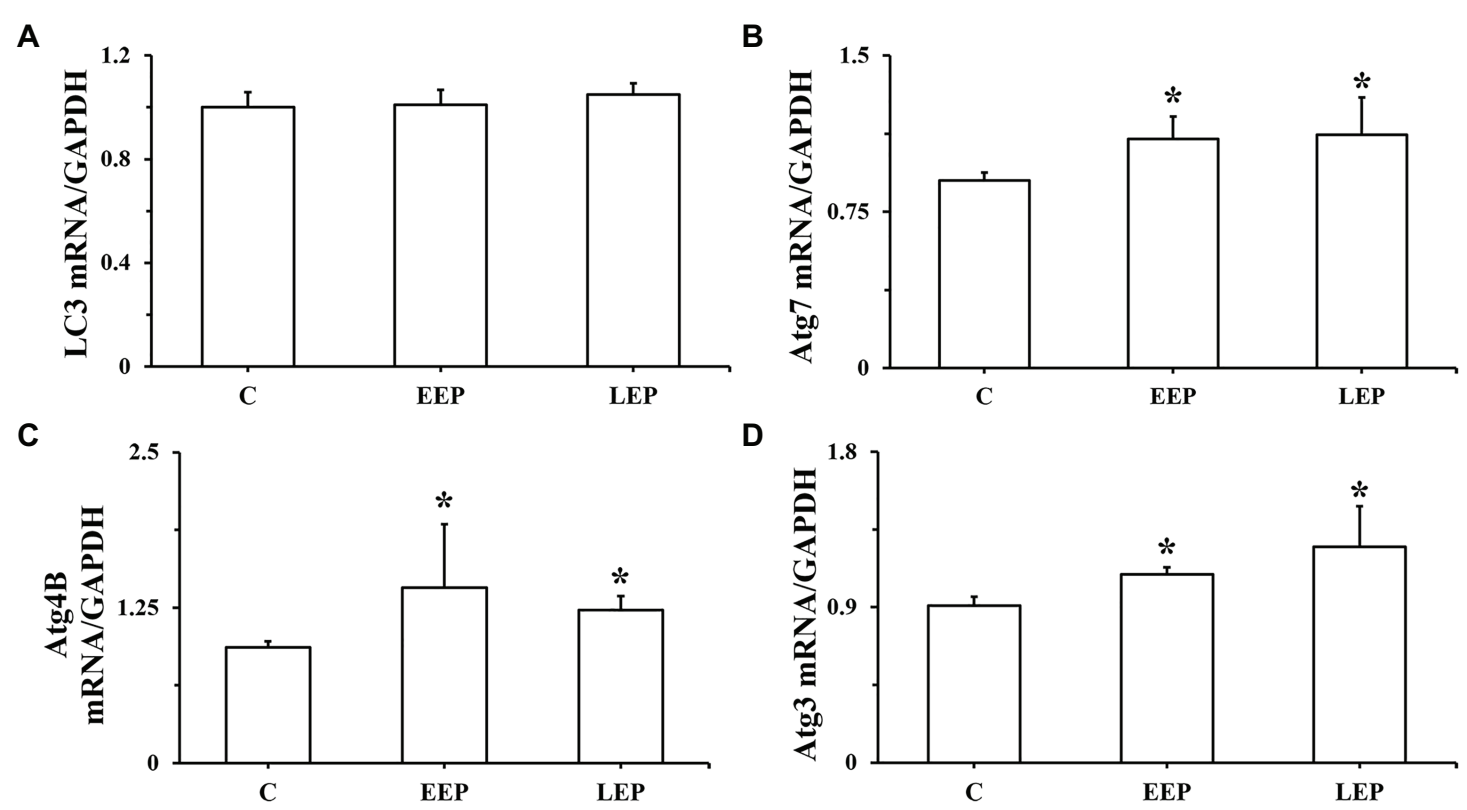
Expression of LC3, Atg7, Atg4B, and Atg3 mRNA during EP. **(A)** LC3 mRNA levels, **(B)** Atg7 mRNA levels, **(C)** Atg4B mRNA levels, and **(D)** Atg3 mRNA levels were determined by real-time PCR (RT-PCR). ^*^p < 0.05 vs. group C.

Real-time PCR results of LC3, Atg7, Atg4B, and Atg3 mRNA are presented in Figures 6A–D. Although there were no significant differences in LC3 mRNA levels in groups EEP and LEP compared with group C, the LC3 mRNA levels were tended to be higher in groups EEP and LEP (*p* > 0.05). In groups EEP and LEP, Atg7, Atg4B, and Atg3 mRNA levels were all significantly upregulated compared with those in group C (*p* < 0.05).

### Expression of LC3 Lipidation-Associated Proteins During PEP

The time to exhaustion in groups EE, EEP + EE, and LEP + EE was 157.80 ± 24.40 min, 166.90 ± 47.93 min, and 197.80 ± 49.43 min, respectively. Although there were no significant differences in the time to exhaustion of group EEP + EE compared with group EE, the time to exhaustion was tended to be higher (*p* > 0.05), suggesting that EEP had the possibility of improving exercise ability of rats. Compared with group EE, the time to exhaustion of group LEP+EE significantly increased (*p* < 0.05), suggesting that LEP could improve the exercise ability of rats.

**Figure 7:**
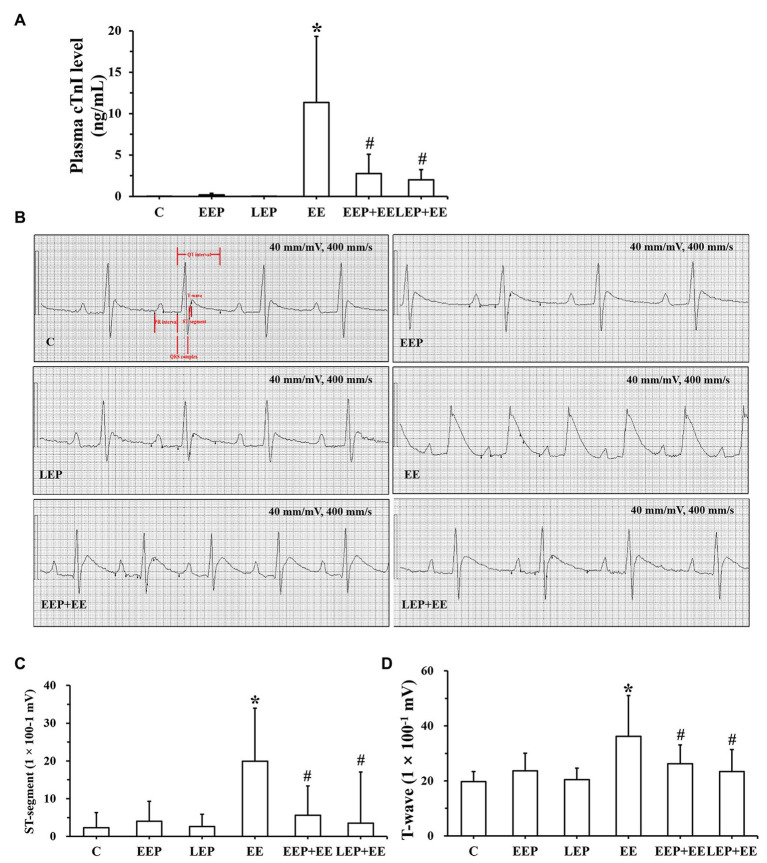
Exercise preconditioning provided cardioprotection to alleviate exhaustive exercise-induced myocardial injury. **(A)** Alterations in plasma cardiac troponin I (cTnI) levels in different groups. **(B)** ECG signals. The ST-segments and T-waves from group C were normal. The ST-segments of group EE were obviously elevated and the T-waves were significantly higher in group EE. **(C)** Results of the ST-segments from ECGs. **(D)** Results of the T-waves from ECGs. ^*^p < 0.05 vs. group C; ^#^*p* < 0.05 vs. group EE.

Data on the plasma cTnI levels from each of these groups are presented in Figure 7A. Compared with group C, there were no significant differences in plasma cTnI levels in groups EEP and LEP (*p* > 0.05), while group EE had significantly higher levels of plasma cTnI (*p* < 0.05). Plasma cTnI levels were robustly decreased in groups EEP + EE and LEP + EE compared with group EE (*p* < 0.05).

Analysis of ECG signals (Figure 7B) demonstrated that the ST-segments and T-waves from group C animals were normal, and there was no obvious change in the ST-segments or T-waves of groups EEP and LEP. The ST-segments of group EE were obviously elevated and the T-waves were significantly higher in group EE. The ST-segments of the ECGs were slightly elevated and the T-waves were slightly higher in groups EEP + EE and LEP + EE. Analysis of the ST-segment and T-wave of ECG signals is shown in Figures 7C,D. This data revealed that compared with group C, the ST-segments of group EE were significantly elevated (*p* < 0.05) and the T-waves of group EE were significantly higher (*p* < 0.05). The ST-segments of groups EEP and LEP were not significantly elevated (*p* > 0.05), and the T-waves of groups EEP and LEP were not significantly higher or inverted compared with those from group C (*p* > 0.05). The ST-segments of groups EEP + EE and LEP + EE were significantly depressed (*p* < 0.05) and the T-waves of groups EEP + EE and LEP + EE were significantly lower (*p* < 0.05) compare with group EE.

**Figure 8:**
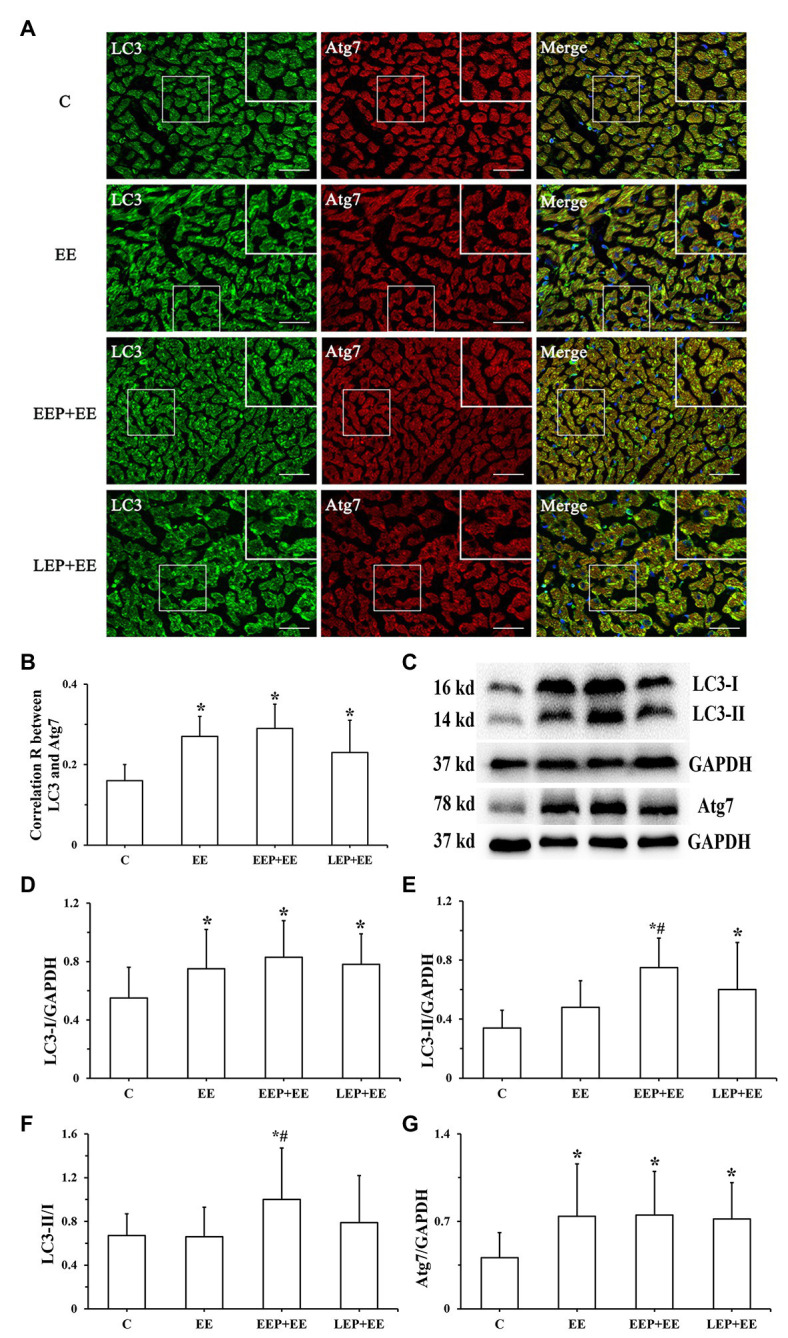
Expression of LC3 and Atg7 levels during EP alleviated myocardial injury induced by exhaustive exercise. **(A)** Immunofluorescent staining of LC3 and Atg7 in cardiomyocytes (bar = 100 μm). LC3 immunofluorescence showed green puncta distributed in the cytoplasm. The immunofluorescence images of Atg7 had red puncta distributed in the cytoplasm, and the merged images yielded yellow puncta. **(B)** Colocalization of LC3 with Atg7 shown as a correlation R. **(C)** Western blot of LC3 and Atg7. **(D)** Western blot analysis of LC3-I in different groups. **(E)** Western blot analysis of LC3-II in different groups. **(F)** Western blot analysis of LC3-II/LC3-I in different groups. **(G)** Western blot analysis of Atg7 in different groups. ^*^p < 0.05 vs. group C; ^#^*p* < 0.05 vs. group EE.

Immunofluorescence colocalization of LC3 with Atg7 in myocardium is shown in Figure 8A. LC3 immunofluorescence images displayed green puncta, including pro-LC3, LC3-I, and LC3-II, distributed in the cytoplasm. The immunofluorescence images of Atg7 had red puncta distributed in the cytoplasm, and the merged images showed yellow overlapping puncta. Statistics on the colocalization of LC3 with Atg7 (Figure 8B) showed that compared with group C, the correlation R was significantly higher in groups EE, EEP + EE, and LEP + EE (*p* < 0.05). There were no significant differences in the correlation R in groups EEP + EE and LEP + EE compared with group EE, while the correlation R in group EEP + EE tended to be higher (*p* > 0.05). Western blot results for LC3 and Atg7 were presented in Figures 8C–G. These results demonstrate that, compared with group C, the LC3-I and Atg7 levels were significantly higher in group EE (*p* < 0.05). Similarly, the LC3-I, LC3-II, and Atg7 levels were significantly higher in groups EEP + EE and LEP + EE compared with group C (*p* < 0.05), and the LC3-II/LC3-I ratio was significantly higher in group EEP + EE compared with group C (*p* < 0.05). The LC3-II and LC3-II/LC3-I levels of group EEP + EE were also significantly elevated compared with group EE (*p* < 0.05).

**Figure 9:**
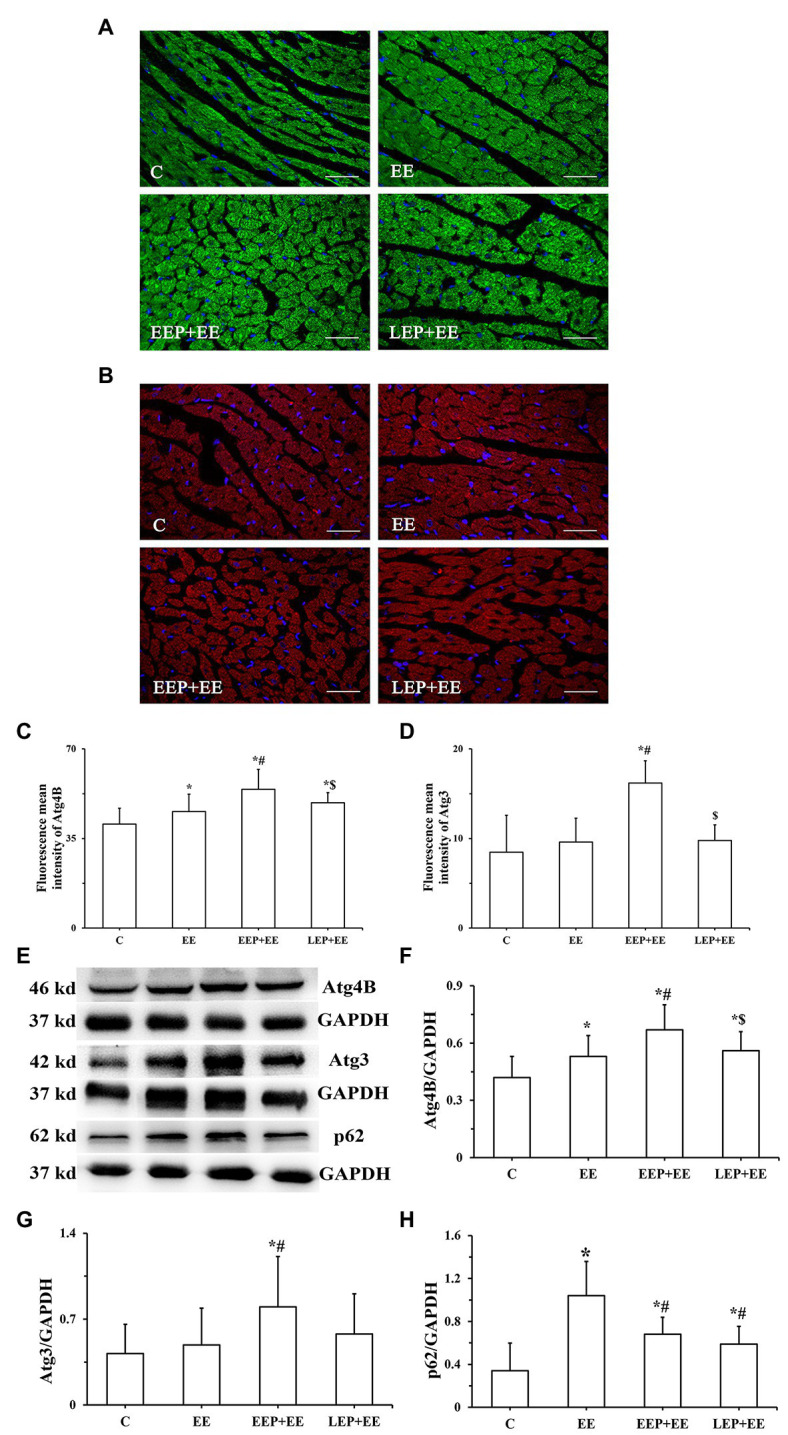
Expression of Atg4B, Atg3, and p62 levels during EP alleviated myocardial injury induced by exhaustive exercise. **(A)** Immunofluorescence staining of Atg4B in cardiomyocytes (bar = 100 μm). Atg4B was stained green and had a scattered distribution in group C. **(B)** Immunofluorescence staining of Atg3 in cardiomyocytes (bar = 100 μm). Atg3 was stained red and had a scattered distribution in group C. **(C)** Fluorescence mean intensity of Atg4B immunofluorescent staining. **(D)** Fluorescence mean intensity of Atg3 immunofluorescent staining. **(E)** Western blot of Atg4B, Atg3, and p62. **(F)** Western blot analysis of Atg4B in different groups. **(G)** Western blot analysis of Atg3 in different groups. **(H)** Western blot analysis of p62 in different groups. ^*^p < 0.05 vs. group C; ^#^*p* < 0.05 vs. group EE; and ^$^*p* < 0.05 vs. group EEP + EE.

The immunofluorescence results (Figures 9A,B) show the expression patterns of Atg4B and Atg3 in myocardium. Atg4B immunoreactive products appeared as green spots, and Atg3 immunoreactive products were presented as red spots, all of which were distributed in the cytoplasm. Analysis of Atg4B and Atg3 immunofluorescence results (Figures 9C,D) showed that compared with group C, the Atg4B fluorescence mean intensity of group EE was significantly higher (*p* < 0.05) in contrast to the Atg3 fluorescence mean intensity of group EE, which tended to be higher (*p* > 0.05). The Atg4B fluorescence intensity was significantly higher in groups EEP + EE and LEP + EE compared with group C (*p* < 0.05). Similarly, the Atg3 fluorescence intensity was significantly higher in group EEP + EE compared with group C (*p* < 0.05). Both the Atg4B and Atg3 fluorescence mean intensity were significantly higher for group EEP + EE compared with the values of group EE (*p* < 0.05). Similarly, our analysis revealed that the Atg4B and Atg3 fluorescence mean intensity were both significantly higher in group EEP + EE compared with group LEP + EE (*p* < 0.05). Western blot results of Atg4B, Atg3, and p62 are shown in Figures 9E–H. They demonstrated that, compared with group C, the Atg4B and p62 levels were significantly higher in groups EE, EEP + EE, and LEP + EE (*p* < 0.05), and the Atg3 levels were significantly higher in group EEP + EE compared with group C (*p* < 0.05). The Atg4B and Atg3 levels of group EEP + EE were also significantly elevated compared with group EE (*p* < 0.05). Similarly, compared with group EE, the p62 levels were significantly decreased in groups EEP + EE and LEP + EE (*p* < 0.05). Meanwhile, the Atg4B levels were robustly elevated in group EEP + EE compared with the level of group LEP + EE (*p* < 0.05).

## Discussion

### EP Induces Myocardial Autophagy Through Intermittent Ischemia-Hypoxia

Exercise can increase the heart rate and cardiac contraction, resulting in increased myocardial oxygen consumption, and relative or absolute myocardial ischemia and hypoxia. EP can lead to myocardial relative or absolute ischemia-hypoxia by repeated high-intensity, intermittent aerobic exercise. C-2R BG staining is a morphological index for detecting early myocardial ischemic-hypoxic injury (Chang et al., 2014). In our study, the degree of myocardial ischemic-hypoxic changes was evaluated as shown by C-2R BG staining. The results showed that, compared with group C, there were different degrees of bright red positive areas in the myocardium of other groups. The IOD and positive area tended to be higher in groups EEP and LEP, and, the IOD and positive area were significantly increased in groups EE, EEP + EE, and LEP + EE (Figure 2). This suggested that IEP, PEP, and exhaustive exercise can all lead to different degrees of myocardial ischemia-hypoxia in rats. Studies have shown that ischemia-hypoxia can induce autophagy under different stress conditions (Gottlieb et al., 2009). Beclin1 is a marker protein for inducing autophagy. In this study, we analyzed the correlation between the IOD and positive area of C-2R BG staining and Beclin1 immunoblotting to explore the relationship between myocardial ischemia-hypoxia and autophagy. The results showed that the IOD and positive area of C-2R BG staining in groups EEP, LEP, EE, EEP + EE, and LEP + EE were positively correlated with the expression of Beclin1 (Figure 3), indicating that the ischemia-hypoxia caused by exercise could increase the level of Beclin1 to enhance the induction of autophagy. This suggested that both EP and exhaustive exercise can induce myocardial autophagy through ischemia-hypoxia (Yuan et al., 2020).

### EP Increases LC3 Lipidation-Associated Proteins Levels to Promote the Formation of Autophagosome

The lipidation of LC3 on autophagosome membrane is an important mechanism for forming autophagosome (Agrotis et al., 2019). Atg4, a cysteine protease, can enable the synthesis of pro-LC3 lipidation and delipidation/deconjugation of lipidated LC3 (Lee et al., 2017). Four Atg4 isoforms (Atg4A to Atg4D) exist in mammals, and Atg4B has obvious specificity and high efficiency for LC3-II. Atg7 is an ubiquitin-activating E1 enzyme that converts LC3 from an immature, cytosolic form to a mature autophagosomal membrane protein with the help of Atg3, an ubiquitin-conjugating E2 enzyme (Pattison et al., 2011). Atg7 bound to LC3-I is important for autophagosomal biogenesis (Pattison et al., 2011). In our current experiments, EP increased Beclin1 levels and induced myocardial autophagy through intermittent ischemia-hypoxia. Additionally, we found that LC3-I, LC3-II levels, the LC3-II/LC3-I ratio, and Atg7 were all significantly increased in parallel, with a significant increase in the combination of LC3 and Atg7 during IEP (Figure 4). Moreover, Atg4B and Atg3 levels were significantly increased during IEP (Figure 5), suggesting that EP could promote the conversion of pro-LC3 to LC3-II by the truncation of Atg4B, activation of Atg7, and conjugation of Atg3, all of which contribute to promote the formation of autophagosome (Li et al., 2018). Autophagy is a dynamic process, which includes not only the formation of autophagosome, but also the transport of autophagy substrate proteins to lysosome where autophagy substrate proteins can be degraded (Lee et al., 2017). p62 is likely to play a role in lysosomal function as an autophagy receptor protein. In this study, our results demonstrated that there was no significant difference in p62 levels during IEP, but the p62 levels tended to be higher (Figure 5), suggesting that IEP might increase receptor protein levels in preparation for enhanced autophagy flux of PEP in the early stage of autophagy.

Smuder et al. (2011) demonstrated that 5 consecutive days of treadmill exercise for 60 min/day at 30 m/min did not significantly upregulate the total expression levels of LC3 mRNA in rat skeletal muscle, but treadmill exercise could significantly increase LC3-II levels. The same phenomenon was observed in this study – EEP could significantly increase LC3-I and LC3-II levels and the LC3-II/LC3-I ratio, but not LC3 mRNA levels. After IEP, LC3 mRNA levels tended to be higher during EEP and LEP. The Atg7, Atg4B, and Atg3 mRNA levels showed the same trend during EEP and LEP, that is, being significantly upregulated (Figure 6). This suggested the transcription levels of LC3 lipidation-associated proteins were positively regulated during EEP and LEP after IEP. Intriguingly, our results demonstrated that the protein levels of Atg4B and Atg3 greatly increased during EEP and LEP, while the protein levels of LC3 and Atg7 only significantly increased during EEP. This suggested the translation levels of LC3 lipidation-associated proteins were positively regulated during EEP and LEP after IEP, and compared with LEP, the rate of translation was more significant during EEP, which might contribute to promote the formation of autophagosome. There was a difference between EEP and LEP in terms of mechanisms of cardioprotection, which was perhaps because EEP could cause modification of existing molecules, whereas newly synthesized proteins could contribute to LEP (Minamino, 2012). In the present study, the positive regulation of transcription and translation level of LC3 lipidation-associated proteins may all be involved in the mechanism of EEP and LEP, while compared with LEP, the regulation of translation level of EEP is more positively to promote autophagy. The total expression levels of LC3 mRNA remained stable in our hands, but the conversion of LC3-I in the cytoplasm to LC3-II in the cytomembrane by lipidation was responsible for enhanced autophagy activity.

### EP Cooperates for Alleviating Exhaustive Exercise-Induced Myocardial Injury by Increasing LC3 Lipidation-Associated Proteins

In this study, we used a rat model of EP prior to exhaustive exercise to evaluate the cardioprotection induced by EP. We used plasma cTnI levels, a unique myocardial injury blood biomarker (Zhang et al., 2019), and C-2R BG staining to comprehensively evaluate exhaustive exercise-induced myocardial injury. In the present study, results showed that plasma cTnI levels and C-2R BG staining had the same trend, which was that they were both significantly decreased during EEP followed by exhaustive exercise and during LEP followed by exhaustive exercise (Figures 2, 7), implying that EP yielded cardioprotection against myocardial injury induced by exhaustive exercise. A single exercise session prior to I/R injury was sufficient to alleviate I/R injury through increasing cardiac output and improving myocardial contractile function (Borges and Lessa, 2015). ECG can be considered a crucial parameter for evaluating cardiac function (Brosnan et al., 2014). The electrophysiological consequences of myocardial ischemia lead to abnormality of the ST-segment and T-wave of an ECG (Tung, 2019). The present study demonstrated that EP gave cardioprotection against exhaustive exercise-induced myocardial ischemic injury by attenuating the ST-segment elevation and T-wave elevation during EEP followed by exhaustive exercise and during LEP followed by exhaustive exercise (Figure 7).

The present study demonstrated a significant increase in myocardial LC3-I, Atg4B levels, the colocalization of LC3 and Atg7, and Atg7 levels, while there were no significantly increases in LC3-II or the LC3-II/LC3-I ratio and Atg3 levels during exhaustive exercise, confirming that exhaustive exercise could not promote the formation of autophagosome (Figures 8, 9). Simultaneously, the levels of p62 were significantly increased during exhaustive exercise in this study (Figure 9). Intracellular LC3-I could not be fully transformed into LC3-II, and accumulation of p62 occurred at the same time, suggesting that the autophagy/lysosomal degradation pathway was inhibited leading to blocked autophagy flux during exhaustive exercise, which could aggravate myocardial injury (Jiang and Mizushima, 2015). Lee et al. (2016) explored the mechanism of autophagy induced by endurance exercise, and their results revealed that acute endurance exercise promoted enhanced autophagy flux (an increase in LC3-II levels, LC3-II/LC3-I ratio, and Atg7 levels, with a reduction in p62 levels), which may be an important mechanism of exercise-induced cardiac health. In this study, we found that LC3-I levels showed a significant increase in the truncation of Atg4B during EEP followed by exhaustive exercise and during LEP followed by exhaustive exercise. Similarly, a significant increase in the conversion of LC3-I to LC3-II through the activation of Atg7 and conjugation of Atg3 during EEP followed by exhaustive exercise and during LEP followed by exhaustive exercise occurred, suggesting that the conversion of pro-LC3 in the cytoplasm to LC3-II in the cytomembrane by lipidation was increased (Figures 8, 9). Meanwhile, EEP and LEP increased the degradation of p62 during exhaustive exercise (Figure 9), resulting in an enhanced autophagy flux, suggesting that the formation of autophagosome and the existence of autophagy flux might play a pivotal role in alleviating myocardial injury induced by exhaustive exercise during EP (Yuan et al., 2020). However, the formation of autophagosome during LEP followed by exhaustive exercise may have been weaker than that during EEP followed by exhaustive exercise due to the lower lipidation of LC3. Since LEP occurred 24 h after IEP, the weakening of autophagy induction led to a decrease in the formation of autophagosome during LEP followed by exhaustive exercise, so the significant increase in LC3 lipidation-associated proteins may have played a more important role in the mechanism of EEP of alleviating myocardial injury induced by exhaustive exercise.

## Conclusion

**Figure 10:**
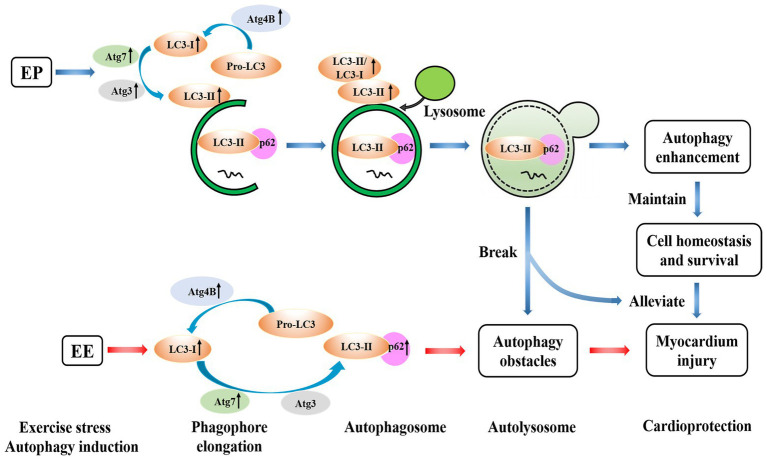
Possible mechanisms of EP alleviating exhaustive exercise-induced injury. EP may promote autophagy to maintain cell homeostasis and survival, which cooperates for cardioprotection of alleviating exhaustive exercise-induced myocardial injury by increasing LC3 lipidation-associated proteins.

Exercise preconditioning can induce myocardial autophagy through intermittent ischemia-hypoxia, and increase LC3 lipidation-associated proteins to promote the formation of autophagosome. Exhaustive exercise may inhibit the formation of autophagosome to cause myocardial injury, whereas EP prior to exhaustive exercise can contribute to alleviate myocardial injury. EP may promote autophagy to maintain cell homeostasis and survival, which cooperates for cardioprotection of alleviating exhaustive exercise-induced myocardial injury by increasing LC3 lipidation-associated proteins (Figure 10). There is a difference between EEP and LEP in terms of the mechanisms of cardioprotection afforded by these respective conditions. The positive regulation of transcription and translation level of LC3 lipidation-associated proteins may all be involved in the mechanism of EEP and LEP, while compared with LEP, the regulation of translation level of EEP is more positively to promote autophagy.

## Data Availability Statement

The original contributions presented in the study are included in the article/supplementary material, further inquiries can be directed to the corresponding author.

## Ethics Statement

The animal study was reviewed and approved by Ethics Committee of Science Research in Shanghai University of Sport, China.

## Author Contributions

S-SP received the National Natural Science Foundation of China and performed the research design and manuscript writing. D-FW, Y-ST, and YH conducted the experiments and performed the data analysis and manuscript writing. All authors contributed to the article and approved the submitted version.

### Conflict of Interest

The authors declare that the research was conducted in the absence of any commercial or financial relationships that could be construed as a potential conflict of interest.

## Abbreviations

EP: Exercise preconditioning
EE: Exhaustive exercise
EEP: Early exercise preconditioning
LEP: Late exercise preconditioning
I/R: Ischemia/reperfusion
IP: Ischemic preconditioning
IEP: Induction of exercise preconditioning
PEP: Protection of exercise preconditioning
LC3/MAP1LC3: Microtubule-associated protein 1 light chain 3
Atg4B: Autophagy-related gene 4B
Atg7: Autophagy-related gene 7
Atg3: Autophagy-related gene 3
C-2R BG staining: Chromotrope-2R brilliant green staining
IOD: Integral optical density
MOD: Mean optical density
cTnI: Cardiac troponin I
